# Protective effects of quercetin against hyperglycemia-induced oxidative stress in hepatic HepG2 cell line

**Published:** 2021

**Authors:** Amir Yarahmadi, Mostafa Moradi Sarabi, Ahmad Sayahi, Fatemeh Zal

**Affiliations:** 1 *Department of Biochemistry, School of Medicine, Shiraz University of Medical Sciences, Shiraz, Iran*; 2 *Transplant Research Center, Shiraz University of Medical Sciences, Shiraz, Iran*; 3 *Department of Biochemistry and Genetics, Lorestan University of Medical Sciences, Faculty of Medicine, Khorramabad, Iran *; 4 *Research Center for Traditional Medicine and History of Medicine, Shiraz University of Medical Sciences, Shiraz, Iran*

**Keywords:** Flavonoid, Quercetin, Antioxidant enzymes, Hyperglycemia, Oxidative stress, HepG2 cells

## Abstract

**Objective:**

Hyperglycemia is a severe consequence of diabetes mellitus (DM). Throughinduction of oxidative stress, it plays a major role in the pathogenesis of several complications in DM. Therefore, new strategies and antioxidants should be implemented inthe treatment of DM. Quercetin is a flavonoid with strong antioxidant capacity found dominantly in vegetables, fruits, leaves, and grains. The current study aimed to investigate quercetin protective effects under D-glucose-induced oxidative stress by assessing antioxidant defense enzymes inHepG2 cells as an *in vitro* model.

**Materials and Methods:**

HepG2 cells were cultured with different concentrations of D-glucose (5.5, 30 and 50 mM) and/or 25 μM quercetin for 48 and 72 hr, respectively. The effect of treatments on cellular integrity, antioxidant enzymes superoxide dismutase (SOD), catalase (CAT), glutathione peroxidase (GPx), glutathione reductase (GR) activity, andcellular levels of glutathione (GSH) and malondialdehyde (MDA) wasdetermined.

**Results:**

D-glucose had various effects on intracellular antioxidant defense atdifferent doses and time-points and quercetin could attenuate oxidative stress and modulate antioxidant defenses.

**Conclusion:**

The results of this study indicated that flavonoid quercetin could be proposed as an agent protecting hepatic HepG2 cells against oxidative stress associated with hyperglycemia.

## Introduction

Hyperglycemia-induced oxidative damage is one of the importantcomplicationsin the pathophysiology of type-2 diabetes mellitus (T2DM) (American Diabetes, 2014 ▶; Roden, 2016 ▶). Hyperglycemia can play an important role in pathophysiological changes that happen during DM through induction of oxidant formation, advanced glycation end products (AGE) generation, hyperosmolarity associated with high glucose, abnormality of sorbitol and myoinositol metabolism, and protein kinase C activation (Evcimen and King, 2007 ▶; Kalousova et al., 2002 ▶). Mechanistically, hyperglycemia generates reactive oxygen species (ROS) that havea pivotal role in D-glucose-induced cellular impairment (Chandrasekaran et al., 2010 ▶; Zal et al., 2014a ▶). ROS consist of superoxide anion radical (O_2˙_^ -^), hydrogen peroxide (H_2_O_2_), hydroxyl radical (OH^-^), and singlet oxygen (^1^O_2_), which are the most reactive oxygen species thatdamage cells during oxidative insult (Hamidi Alamdari et al., 2020 ▶; Nimse and Pal, 2015 ▶). Antioxidants that neutralize ROS to prevent cellular components from oxidative insult, can be divided into enzymatic and non-enzymatic antioxidants. Enzymatic antioxidants, including superoxide dismutase (SOD), catalase (CAT), glutathione peroxidase (GPx) and glutathione reductase (GR) and non-enzymatic antioxidants (e. g. GSH, vitamin A, C, and E, and bioflavonoids) have been implicated in the protection against ROS (Di Luzio and Hartman, 1967 ▶; Kazemi et al., 2015 ▶; Yazdi et al., 2020). The enzyme SODis located in both cytosol and mitochondria and catalyzes conversion of O_2˙_^-^ into O_2_ and H_2_O_2 _by using metal cofactors copper (Cu), zinc (Zn), and manganese (Mn), respectively. In the peroxisome of cells, the enzyme CAT converts H_2_O_2_ to H_2_O and O_2_. GPx is another enzyme responsible for dissociation of H_2_O_2 _into H_2_O via GSH in both cytoplasm and extracellular fluid of allhuman tissues. GR functions by reducing oxidized GS-SG into its active form GSH which is necessary for the action of GPx (Nimse and Pal, 2015 ▶). Antioxidants tightly regulate the cellular redox balance and can delay or inhibit oxidative damage by converting free radicals to non-toxic products (Amin et al., 2020 ▶; Mashhoody et al., 2014 ▶; Weydert and Cullen, 2010 ▶). Flavonoids are employed as the most attractive agents among the antioxidants (Lodhi et al., 2016 ▶; Saboonchian et al., 2014 ▶). They have different actions including antiallergic, anti-inflammatory, antiseptic, and antioxidant, and exert limited toxicity (Choi et al., 2017 ▶; Srivastava et al., 2016 ▶). However, potent antioxidant activities of flavonoids are due to the high redox potential that allows them to act as reducing agents for reducing and scavenging free radicals (Anand David et al., 2016 ▶; Razali et al., 2015 ▶; Srivastava et al., 2016 ▶). Quercetin, primarily known for its prominent antioxidant capacity, is one of the most abundant flavonoids widely found in various food products, fruits, and vegetables (Ader et al., 2000 ▶; Mulholland et al., 2001 ▶). Quercetinhas been investigated for its beneficial effects such as the prevention of cell death and oxidant damage (Yarahmadi et al., 2017 ▶; Zizkova et al., 2017 ▶). It is also famous for protecting DNA from oxidative damages of O_2˙_^-^, H_2_O_2_, and OH^-^ on DNA oligonucleotides. It has been suggested that quercetin has a beneficial impact on diabetes by reducing hyperglycemia-induced oxidative stress in hepatic tissue (Chis et al., 2016 ▶; Yarahmadi et al., 2018 ▶). Moreover, many studies have indicated that the antioxidant capacity of quercetin is stronger than other phenolic compounds (Bai et al., 2013 ▶). Given that hyperglycemia injures the cells through oxidative stress in diabetes and considering the role of quercetin as a potent antioxidant, the present study aimed to investigate potential protective effects of quercetin against oxidative stress induced by D-glucose atdifferent doses and time-points in HepG2 cells *in vitro*. 

## Materials and Methods

**Chemicals**

Quercetin and D-glucose were purchased from Sigma Chemical Co. (St. Louis, MO, USA); Roswell Park Memorial Institute Medium (RPMI), fetal bovine serum (FBS), penicillin, and streptomycin were obtained from Gibco-BRL (Paisley, UK). BSA total protein assay kit was purchased from Bio-Rad (Hercules, California, USA). Trypsin was from BDH, England. Potassium chloride, and potassium dihydrogen orthophosphate were from Fluka, England. GR, tert-butyl hydroperoxide (t-BuOOH), bovine serum albumin (BSA), nicotinamide adenine dinucleotide phosphate (NADPH), Triton X-100, ethylenediaminetetraacetic acid (EDTA), and dimethyl sulfoxide (DMSO) were purchased from Sigma Chemical Co. (Poole, Dorset, UK).

**Cell culture and treatment**

The human HepG2 cell line was acquired from the National Cell Bank of Iran (NCBI, Pasteur Institute, Tehran). Then, cells were cultured in RPMI 1640 medium containing 10% heat-inactivated FBS, supplemented with 100 IU/ml penicillin and 100 µg/ml streptomycin in a humidified atmosphere containing 5% CO_2_ at 37ºC. All experiments were done using cells at 70% confluences (Zal et al., 2020 ▶). Cells were then grown and divided into 6 groups as follows:the first group includedmedia containing 5.5 mM D-glucose (as a normal concentration and served as normal control cells), the second group includedD-glucose 30 mM, the third group includedD-glucose 50 mM (as thehigh concentration), and the fourth group was treated with 25 μM quercetin, the fifth group includedD-glucose 30 mM + quercetin (25 μM) and finally, the sixth group was treated with D-glucose 50 mM + quercetin (25 μM). The vehicle for the quercetin was ethanol, and a separate group was treated with ethanol (0.3%), and since it did notshow significant differences with control, was omitted from analysis. We performed all treatments in quadruplicate foreach group. Treated cells were incubated at 37ºC with 5% CO_2_. The cell lysate was collected after 48 and 72 hr treatment and stored at -20ºC until use. Notably, the pH of the culture medium was not affected by addition of D-glucose or quercetin. 

**Cell viability assay**

To assess the viability of cells treated with different concentrations of D-glucose or quercetin, an assay was carried out using 3-(4, 5-dimethylthiazol- 2-yl)-2, 5 diphenyltetrazolium bromide (MTT) as previously described by Mosmann(Mosmann, 1983). First, 12×10^3^ cells/well in a 96-well plate were incubated with 5.5, 30 and 50 mM D-glucose and/or 25 μM quercetin for 48 and 72 hr, respectively at 37ºC. Then, cells were incubated with MTT (0.5 mg/ml) dissolved in serum-free medium. After 3.5 hr incubation, 100 μl DMSO was added to dissolve the formazan crystals, and then, absorbance was determined at 570/650 nm wavelength using an ELISA reader (Bio Rad, USA). Cell viability was determined as the ratio of absorbance of treated cells to that of untreated cells that served as a control.

**Measurement of Cu, Zn-SOD activity**

We measured the activity of Cu, Zn-SOD in HepG-2 cell lysate based on the procedure that was described by Misra and Fridovich (Misra and Fridovich, 1972 ▶). This method relies on assessing inhibition of adrenaline auto-oxidation to adrenochrome by enzyme SOD in alkaline pH 10.2 at 480 nm. First, we drewthe standard curve of SOD by plotting the amount of inhibition of adrenaline oxidation against different concentrations of SOD. Each unit of SOD enzyme is the amount of enzyme that inhibits 50% adrenaline oxidation, and SOD enzyme activity isreported as unit/mg protein of the HepG2 cell lysate.

**Measurement of CAT activity**

For determination of CAT activity, we used a method that was previously described by Aebi (Aebi, 1984 ▶). In this method, we spectrophotometrically assayed decomposition of H_2_O_2_ to H_2_O and O_2 _by CAT.Enzyme activity isshown as mmol H_2_O_2_ consumed/min per mg HepG2 cell lysate protein by a molar absorptivity of 43.6 M/cm.

**Measurement of GPx activity**

GPx activity was measured bythe process described by Fecondo and Augustey based on monitoring continuous substitution of reduced GSH from oxidized form (G-S-S-G) in the presence of enzyme glutathione reductase. Besides, we used disodium (Na_2_) salt of reduced nicotinamide adenine dinucleotide phosphate for the assessment of GPx activity with minor modifications (Mostafavi-Pour et al., 2008 ▶). GPx enzyme activity in the HepG2 cell lysate is shownas μmol of NADPH oxidized/min/mg of cell protein using a molar absorptivity of 6.22×10^6 ^M/cmfor NADPH. One unit of GPx is shownas U/mg of HepG2 cell protein.

**Measurement of GR activity**

The enzyme GR activities were measured using the method of Racker and Carlberg (Carlberg and Mannervik, 1985 ▶; Racker, 1955 ▶). For determination of GR activity, 60 μM buffer, 5 mM EDTA (pH 8.0), 0.033 M GS-SG, 2 mM NADPH, and a sample of HepG2 cell lysate ata final volume of 1000 μl, were used. A reduction in absorbance shows oxidation of NADPH through reduction of GS-SG by the activity of enzyme GR in the HepG2 cell lysate which was monitored spectrophotometrically at 340 nm wavelength for 3 min. Finally, the results arereported through a molar absorptivity of 6.22×10^6 ^M/cmfor NADPH. One unit of GR is defined as U/mg HepG2 cell protein.

**Determination of reduced GSH**

Assessment of reduced GSH using 5, 5-dithio-bis (2-nitrobenzoic) acid (DTNB), was performed using Ellman’s method with some modifications (Mashhoody et al., 2014 ▶; Zal et al., 2014b ▶). We used 1mM solutions of reduced GSH to draw a standard curve. GSH amount was assayed in the HepG2 cell lysate. For measurement of GSH level, 0.5 ml of DTNB (0.001 M) solution was added to 2.3 ml potassium phosphate buffer (0.2 M, pH 7.6) plus 0.2 ml of HepG2 cell lysate. The absorbance of the solution was read after 5 min at 412 nm.

**Determination of MDA**

MDA as a marker of lipid peroxidation was estimated according to a colorimetric method. Briefly, 100 μl of the HepG2 cell lysate was added to 400 μl TBA reagent containing 0.375% TBA, 15% trichloroacetic acid, and 0.25 mol/lHCl. The mixture was boiled in a water bath at 95ºC for 30 min and after fast cooling, it was centrifuged at 8000×g for 15 min at 4ºC. The absorbance of the pink color supernatant was measured at 532 nm. MDA concentration was calculated using tetraethoxypropane (TEP) as standard and expressed as nmol/mg protein.

**Measurement of total protein**

Protein content was determined using BSA as a standard with BSA total protein assay kit from Bio-Rad according to the manufacturer protocols.

**Statistical analysis**

GraphPad Prism version 6.01 (GraphPad Software, San Diego, CA, USA) and SPSS18 software (SPSS, Chicago, IL, USA) were used for group comparison. The data arepresented as mean±SD and analyzed usingone-way analysis of variance (one-way ANOVA) followed by Tukey’s multiple comparison test. A p-value≤0.05 was used as the level of signiﬁcance. Mean±SD valuesare the representative of four independent experiments.

## Results

**Effect of D-glucose and quercetin on the viability of HepG2 cells**

**Figure 1:**
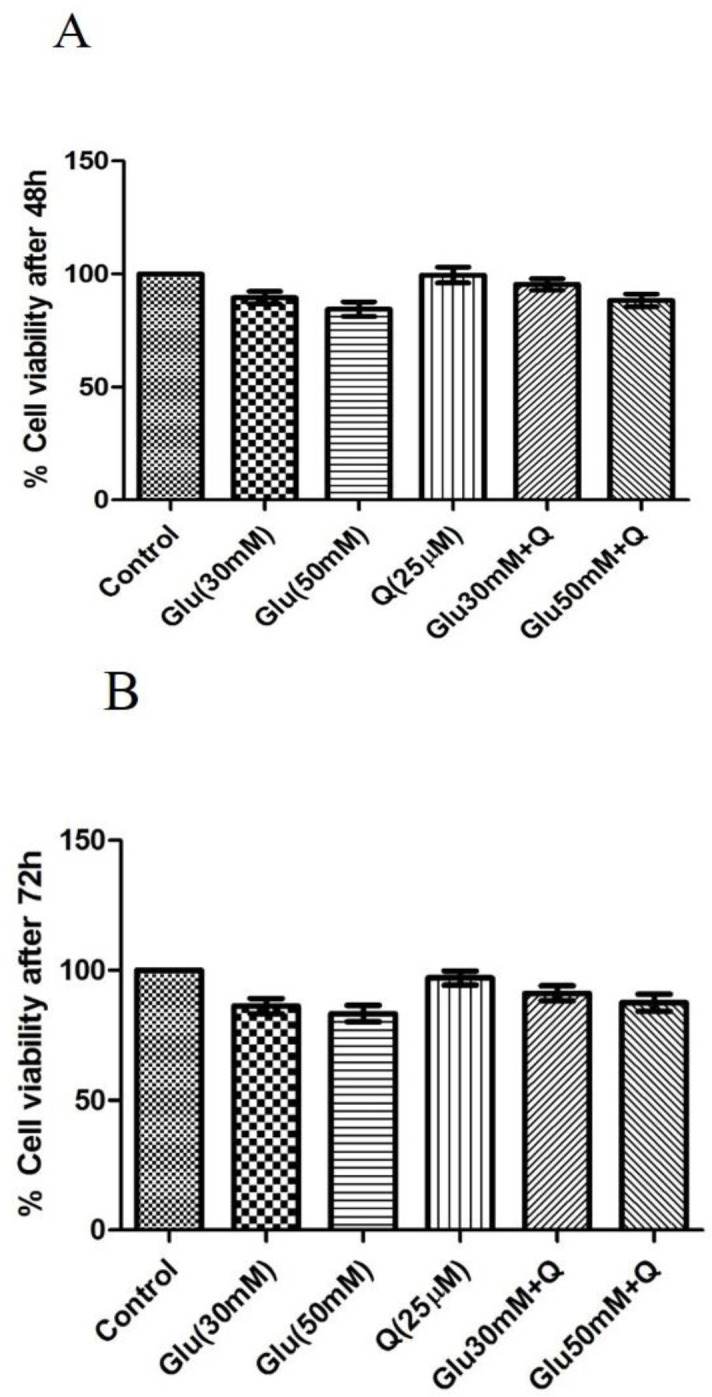
Effect of D-glucose and/ or quercetin on the viability of HepG2 cells after 48 (A) and 72 (B) hr treatment using the MTT assay. Data are presented as mean±SD (n=4). All data are presented as a percentage with respect to control (100% cell viability). Glu: D-glucose; Q: Quercetin; and MTT: 3-(4, 5-dimethylthiazol-2-yl)-2, 5 diphenyltetrazolium bromide

Figure 1 (A and B) showsthat viability was not affected when HepG2 cells were incubated with 30 and 50 mM D-glucose with or without quercetin for 48 and 72 hr, respectively (85-90% viability).

**Effect of D-glucose and quercetin on SOD activity**

**Figure 2:**
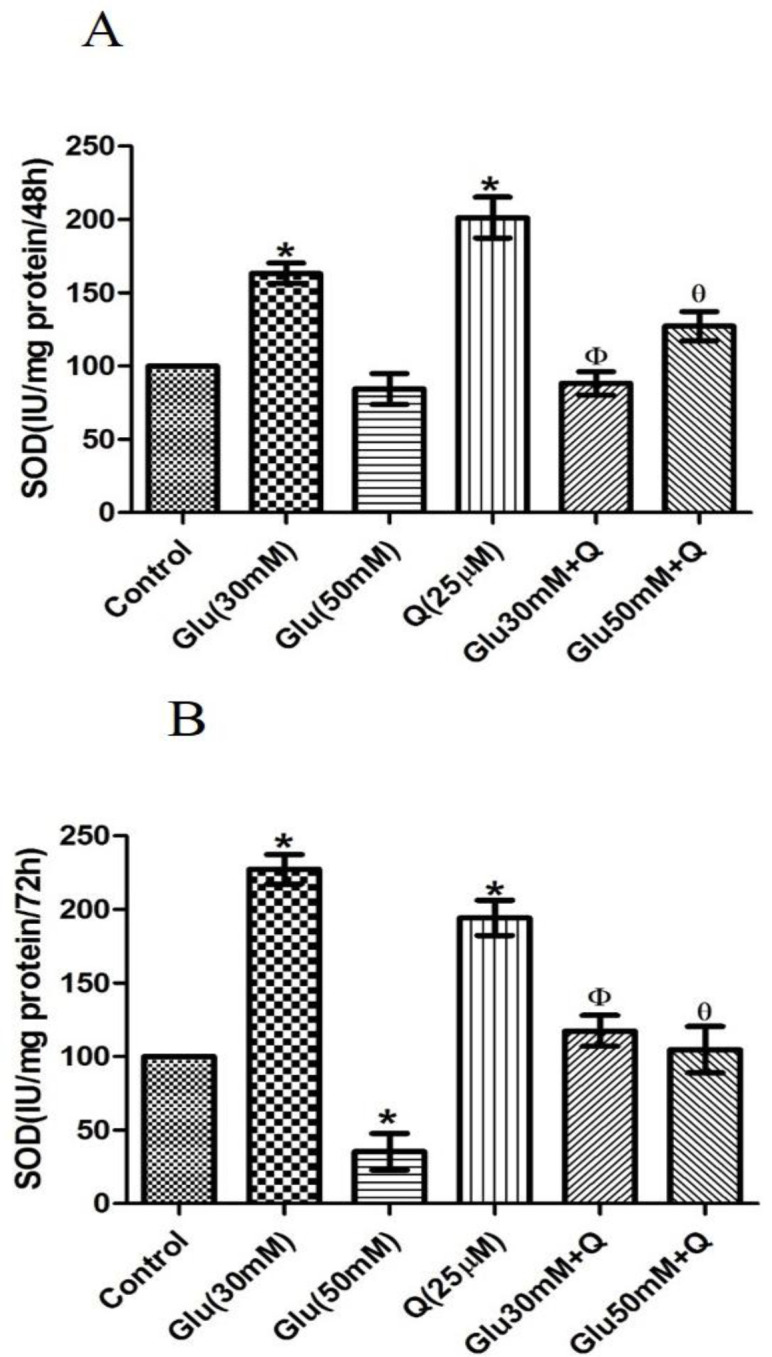
Effect of D-glucose and/ or quercetin on SOD activity in HepG2 cells after 48 (A) and 72 (B) hr treatment. Results are given as mean±SD (n=4). *shows a significant difference compared to the respective control group (p<0·05); Φshows asignificant difference compared to 30 mM D-glucose-treated group (p<0·05); Ɵ shows asignificant difference compared to 50 mM D-glucose-treated group (p<0·05). Glu: D-glucose; Q: Quercetin; and SOD: Superoxide dismutase

The effect of D-glucose on SOD activity was different atdifferentconcentrations and time-points. As shown in Figure 2, after both 48 and 72 hr, 30 mM D-glucose significantly increased SOD activity, but 50 mM D-glucose decreased it though itwas significant only after 72 hr of treatment with 50 mM D-glucose (p<0.05). The combinations of quercetin and30 mM D-glucose significantly decreased the level of SOD activity compared to the corresponding values in HepG2 cells treated with 30 mM D-glucose, while combinations of quercetin and 50 mM D-glucose significantly increased the level of SOD activity compared to the corresponding values at 50 mM D-glucose concentration (p<0.05).

**Effect of D-glucose and quercetin on CAT activity**

**Figure 3:**
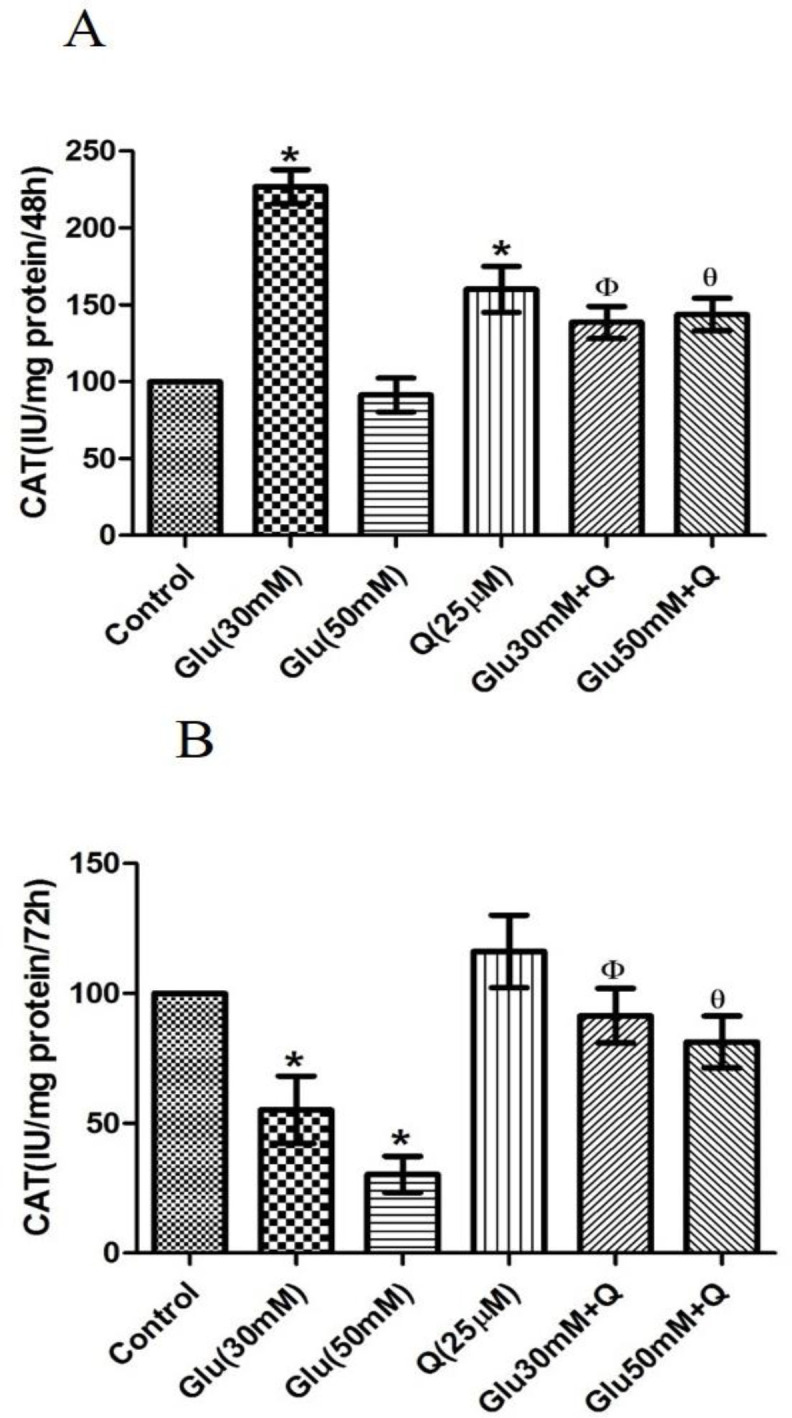
Effect of D-glucose and/ or quercetin on CAT activity in HepG2 cells after 48 (A) and 72 (B) hr treatment. Results are given as mean±SD (n=4). * shows asignificant difference compared to the respective control group (p<0·05); Φ shows asignificant difference compared to 30 mM D-glucose treated group (p<0·05); Ɵ shows asignificant difference compared to 50 mM D-glucose treated group (p<0·05). Glu: D-glucose; Q: Quercetin; and CAT: Catalase

As shown in Figure 3, after 48 hr, a 2.2 fold increase in CAT activity was observed in HepG2 cells treated with 30 mM D-glucose as compared to normal control cells, but after 72 hr, 44.7% decrease in CAT activity in30 mM D-glucose-treated group occurred (p<0.05). Also, 50 mM D-glucose significantly decreased CAT activity by about 69% after 72 hr as compared to the normal control cells (p<0.05). Atboth 48 and 72 hr time-points, quercetin could improve these conditions compared to the corresponding values in HepG2 cells treated with 30 and 50 mM D-glucose, respectively (p<0.05). Quercetin-treated cells showed an increase in CAT activity, which was significant only after 48 hr of treatment as compared to the normal control cells (p<0.05).

**Effect of D-glucose and quercetin on GPx activity**

**Figure 4:**
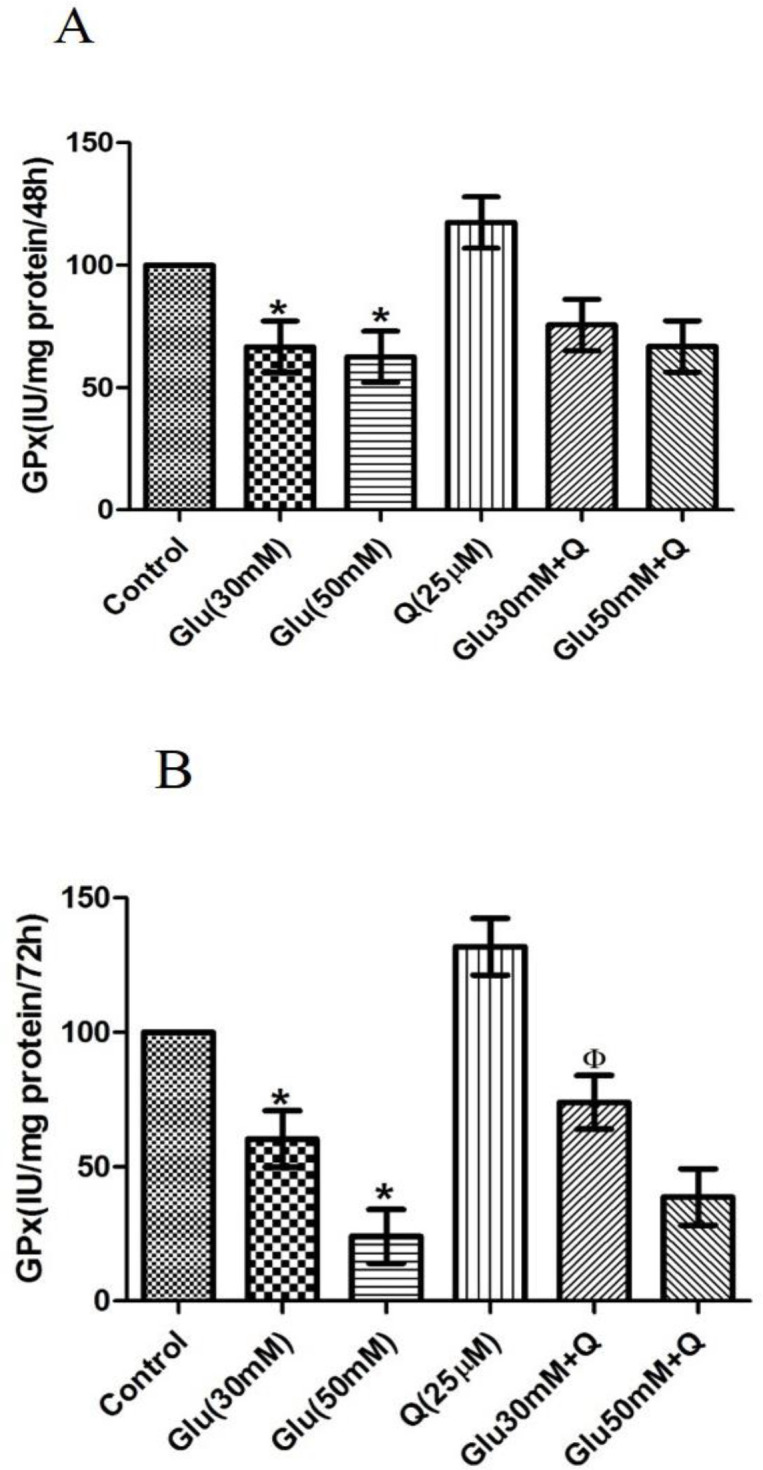
Effect of D-glucose and/ or quercetin on GPx activity in HepG2 cells after 48 (A) and 72 (B) hr treatment. Results are given as mean±SD (n=4). *shows asignificant difference compared to the respective control group (p<0·05); Φ shows a significant difference compared to 30 mM D-glucose treated group (p<0·05).Glu: D-glucose; Q: Quercetin; and GPx: Glutathione peroxidase

Our data showed that treatment of HepG2 cells with 30 and 50 mM D-glucose for 48 hr resulted in significant 33 and 38% decreases in GPx activity, respectively (p<0.05) (Figure 4). However, after 72 hr, this reduction was 40 and 75.7% as compared to the control cells, respectively (p<0.05) (Figure 4). Furthermore, incubation of HepG2 cells with quercetin and quercetin plus 30 and 50 mM D-glucose caused an increase in GPx activity as compared to the control group, but this increase was significant only at 30 mM D-glucose + quercetinafter 72 hr.

**Effect of D-glucose and quercetin on GR activity**

**Figure 5:**
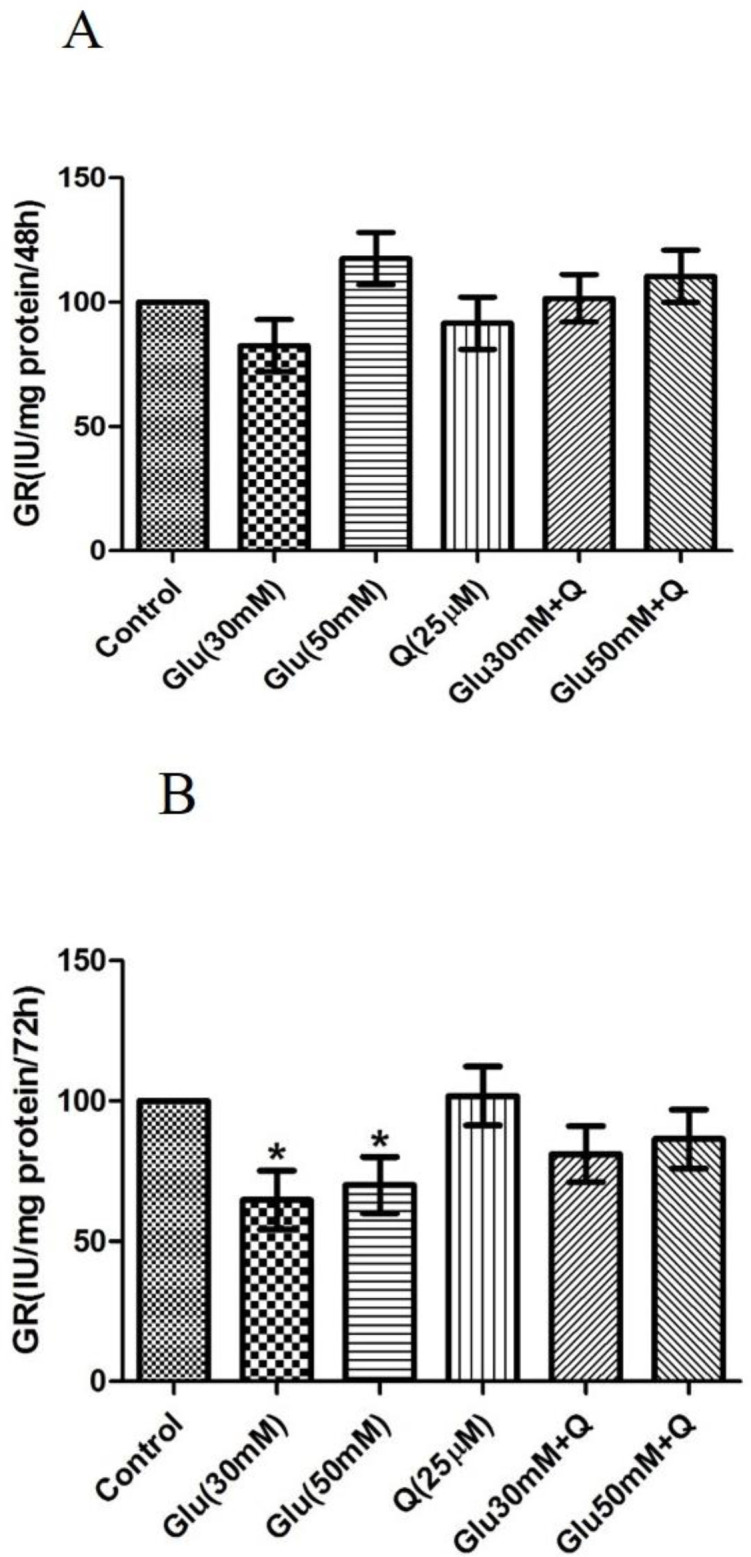
Effect of D-glucose and/ or quercetin on GR activity in HepG2 cells after 48 (A) and 72 (B) hr treatment. Results are given as mean±SD (n=4). * shows a significant difference compared to the respective control group (p<0·05).Glu: D-glucose; Q: Quercetin; and GR: Glutathione reductase

As shown in Figure 5, after 48 hr, we found no significant differences in GR activity in D-glucose-treated HepG2 cells with or without quercetin as compared to the control group. However, after 72 hr, 30 and 50 mM D-glucose significantly decreased GR activity, and quercetin could not overcome this reduction (p<0.05) (Figure 5).

**Effect of D-glucose and quercetin on GSH level in HepG2 cells**

**Figure 6:**
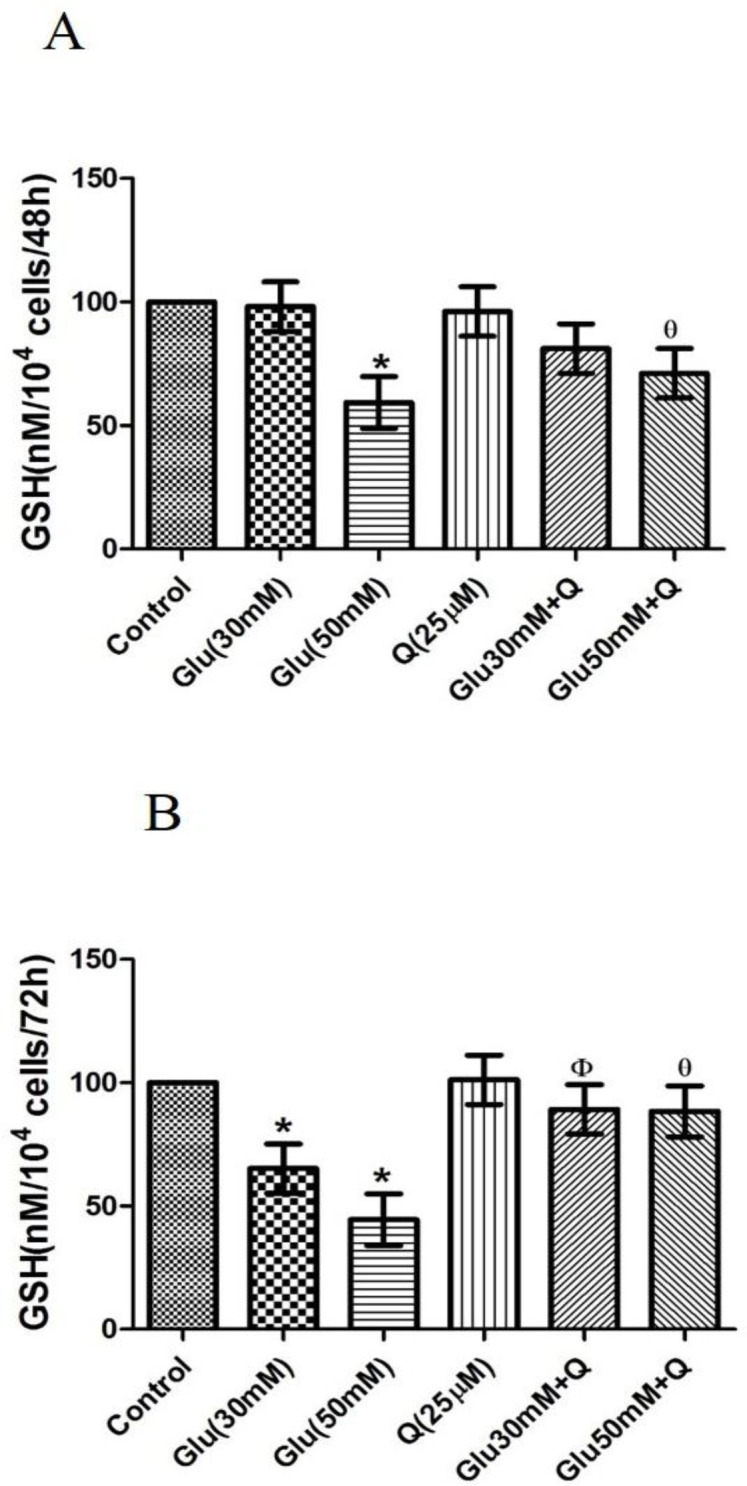
Effect of D-glucose and/ or quercetin on GSH level in HepG2 cells after 48 (A) and 72 (B) hr treatment. Results are given as mean±SD (n=4). * shows asignificant difference compared to the respective control group (p<0·05); Φ shows asignificant difference compared to 30 mM D-glucose treated group (p<0·05); Ɵ shows asignificant difference compared to 50 mM D-glucose treated group (p<0·05). Glu: D-glucose; Q: Quercetin; and GSH: Glutathione

Figure 6 shows that the level of GSH was significantly decreased by 41% in HepG2 cells treated with 50 mM D-glucose after 48 hr, as compared to the control group (p<0.05). 

Also, Figure 6 demonstrates that after 72 hr, 30 and 50 mM D-glucose significantly decreased GSH by 34.4 and 55.5%, respectively, in treated HepG2 cells in comparison to the control group (p<0.05).

After 48 hr incubation of HepG2 cells treated with quercetin plus 50 mM D-glucose, a significant increase in GSH level occurred compared to 50 mM D-glucose-treated cells (p<0.05). 

Additionally, our data indicated that after 72 hr, incubation of HepG2 cells with quercetin plus 30 and 50 mM D-glucose caused a significant increase in GSH level compared to the corresponding 30 and 50 mM D-glucose treated HepG2 treated cells, respectively (p<0.05) (Figure 6).

**Effect of D-glucose and quercetin on MDA level in HepG2 cells**

**Figure 7:**
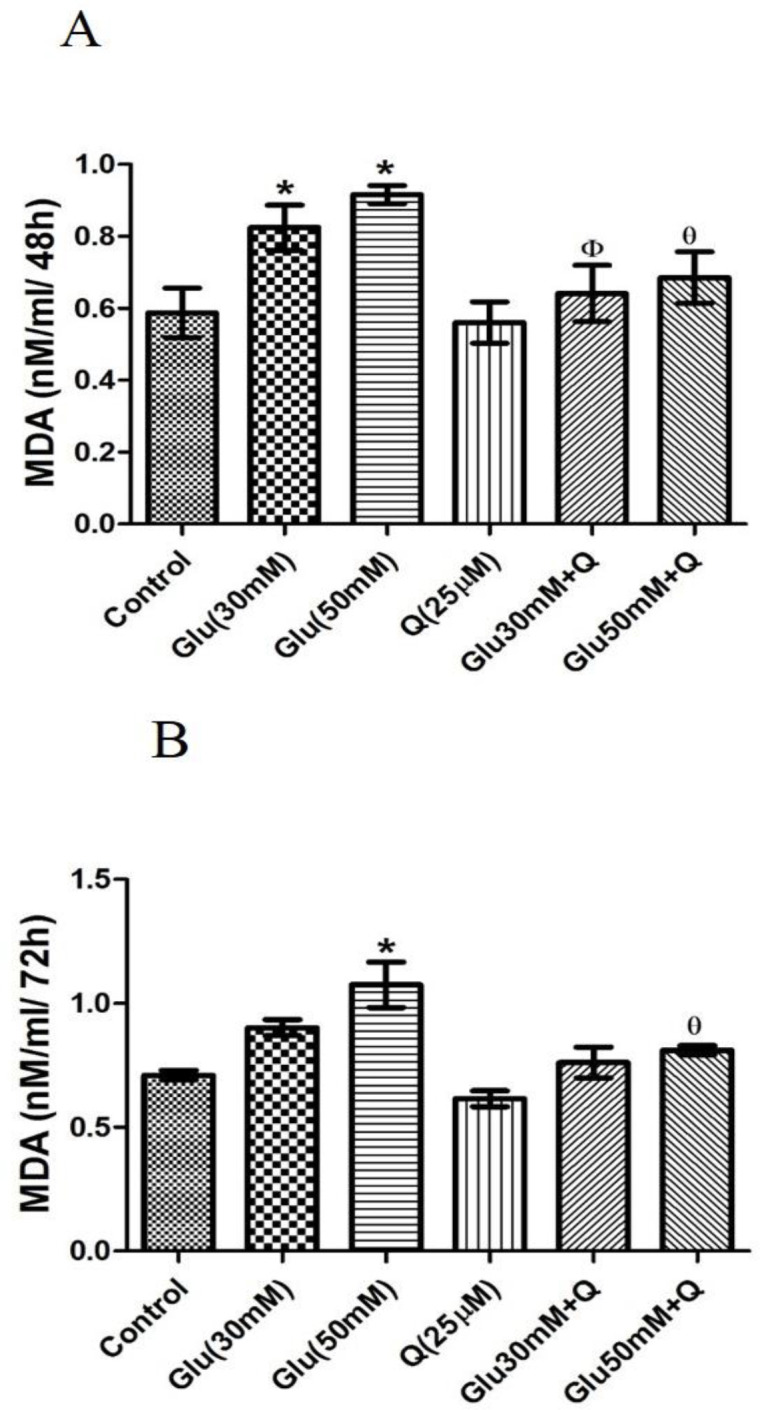
Effect of D-glucose and/ or quercetin on MDA level in HepG2 cells after 48 (A) and 72 (B) hr treatment. Results are given as mean±SD (n=4). * shows asignificant difference compared to the respective control group (p<0·05); Φ shows asignificant difference compared to 30 mM D-glucose treated group (p<0·05); Ɵ shows asignificant difference compared to 50 mM D-glucose-treated group (p<0·05). Glu: D-glucose; Q: Quercetin; and MDA: Malondialdehyde

As shown in Figure 7, 48 hr treatment of HepG2 cells with 30 and 50 mM D-glucose resulted in 40 and 56% increases in the level of MDA as a marker of lipid peroxidation compared to the control group (p<0.05). 

Moreover, quercetinproduced a significant decrease in the level of MDA in cells treated with 30 and 50 mM D-glucose (p<0.05).

Our results also showed that 72hr treatment of HepG2 cells with 50 mM D-glucose significantly increased the level of MDA to about 41%, and quercetin significantly decreased the MDA level in 50 mM D-glucose-treated cells (p<0.05) (Figure 7).

## Discussion

The current study aimed to investigate the effects of quercetin on hyperglycemia and hyperglycemia-induced disruption of antioxidant defense system in HepG2 cells. There are several studies indicating that HepG2 cells are a well-characterized cell line to study hyperglycemia *in vitro* (Chandrasekaran et al., 2010a ▶; Alia et al., 2006). 

Chandrasekaran et al. used 30 and 50 mM D-glucose for 72 hr to show toxic effects of hyperglycemia on HepG2 cells (Chandrasekaran et al., 2010 ▶). Similarly, Cordero-Herrera et al. used different D-glucose concentrations ranging from 20-60 mM for 24 hr, to assess hyperglycemia effects in human HepG2 cells (Cordero-Herrera et al., 2014 ▶). Moreover, Buranasin et al. used 25 and 50 mM D-glucose for 72 hr to create hyperglycemic conditions in human gingival fibroblasts (Buranasin et al., 2018 ▶).

Our results showed that D-glucose atdifferent concentrations and time-points had different effects on cellular antioxidant enzyme patterns, which may be explained by this fact that the amount and time of the exposure to produce oxidative conditions may influence the antioxidant enzyme defense capacity. Oxidative stress resulted from higher concentrations and longer time periods of D-glucose exposure (50 mM for 72 hr) mayovercomethe antioxidant enzyme defenses capacity in HepG2 cells compared to the cells treated with 30 mM D-glucose for 48 hr.

ROS are generated atlow quantities during normal cell metabolism. However, ROSoverproduction has been observed in many diseases, such as diabetes and many types of cancers (Matsuda and Shimomura, 2013). A high level of D-glucose promotes generation of ROS during diabetes that plays a crucial role in D-glucose-induced cellular dysfunction and oxidative stress (Chandrasekaran et al., 2010b ▶). Our data demonstrated that treatment of HepG2 cells with D-glucose evoked a notable increase in SOD activity, and quercetin significantly counterbalancedit, which is compatible with the study of Ceriello et al. showing that hyperglycemia increased the activity and mRNA expression of Cu, Zn-SOD and Mn-SOD in human endothelial cells (Ceriello et al., 1996 ▶). Similarly, Weidig et al. showed that incubation of rat coronary endothelial cells with 22 mM D-glucose significantly increased Cu, Zn-SOD activity and free radical scavenger mercaptopropionylglycine reduced it to that of the control group (Weidig et al., 2004 ▶). Our results showed a significant decrease in SOD activity after 72 hr of treatments with 50 mM D-glucose, but quercetin significantly raised it. This is compatible with Uchimura et al. study where they showed significantly lower levels of Cu, Zn-SOD and Mn-SOD activities in neutrophils and lymphocytes of patients with diabetes mellitus compared to healthy controls, which can be explained by this theory that adaptive or toxicity responses may depend on time and concentration of exposure (Uchimura et al., 1999). In our experiment, the activity of CAT significantly increased after 48 hr exposure to 30 mM D-glucose and quercetin significantly reduced this increase compared to the corresponding value. However, after 72 hr treatment, CAT activity significantly decreased in both 30 and 50 mM D-glucose-treated cells. These results are compatible with the Dave et al. study in which hyperglycemia decreased CAT and GPx activity in both types of diabetes (Dave and Kalia, 2007 ▶). Consistent to our study, Alam et al. showed that CAT, SOD, glutathione-S-transferase and GSH levels were significantly increased in type-2 diabetic mice treated with quercetin for three weeks compared to normal controls (Alam et al., 2014). The current study demonstrated that treatment of HepG2 cells with different concentrations of D-glucose caused a significant reduction in GPx activity after both 48 and 72 hr respectively, and quercetin increased GPx activity, but it was significant only in HepG2 cells that were treated with 30 mM D-glucose plus quercetin for 72 hr. Thesedata are compatible with the study conducted by Amaral et al. They showed that hyperglycemia reduces GPx activity in testicular cells isolated fromdiabetic rats (Amaral et al., 2006). Moreover, Jeonget al. showed that consumption of quercetin bytype 2 diabetic db/db mice, increases GPx activity in the liver after six weeks of treatment (Jeong et al., 2012). 

Furthermore, our results showed that GR enzyme activity and levels of GSH decreased in D-glucose-treated HepG2 cells, especially in a concentration- and time-dependent manner. It may be primarily due to intracellular oxidation and increaseof oxidative stress in cellular environments (Chandrasekaran et al., 2010b ▶; Hall, 1999). Moreover, we observed that quercetin treatment of HepG2 cells prevents D-glucose-induced oxidation effects; our results were comparable to those of other studies which demonstrated that quercetin exhibits a preventative effect against hyperglycemia-induced mitochondrial impairment in liver cells (Zou et al., 2014). 

In conclusion, quercetin can attenuate oxidative stress caused by hyperglycemia and protect human HepG2 cells by modulating antioxidant defense system. However, further pharmacological studies are required to establish its beneficial effects in patients with disrupted oxidative balance such as diabetes mellitus.
